# Mimetic Butterflies Introgress to Impress

**DOI:** 10.1371/journal.pgen.1002802

**Published:** 2012-06-21

**Authors:** Marcus R. Kronforst

**Affiliations:** 1 FAS Center for Systems Biology, Harvard University, Cambridge, Massachusetts, United States of America; 2 Department of Ecology and Evolution, University of Chicago, Chicago, Illinois, United States of America; Stanford University School of Medicine, United States of America

The extent to which hybridization and the resulting interspecific gene flow (introgression) contribute to adaptation is a matter of great debate. On the one hand, fertile hybrids have the potential to transfer beneficial alleles between species [1] or even to spawn new species [2]. On the other hand, hybrids tend to have reduced fitness relative to parental species [3], which will often make them an evolutionary dead end. Only a handful of examples of adaptive introgression are known, such as recent evidence for the transfer of warfarin resistance between mouse species [4]. However, one place where introgression of beneficial alleles could be common is in adaptive radiations [5]. These explosions of phenotypic and species diversity may be just the place to look for adaptive introgression because they often contain closely related, hybridizing species, and introgression could provide the raw genetic material for their exceptional rates of diversification. Two new papers, one in this issue of *PLoS Genetics* [6] and another in *Nature* [7], provide long-awaited evidence for a direct role of introgression in fueling a particularly striking adaptive radiation, the mimetic wing pattern radiation of *Heliconius* butterflies.

The Neotropical genus *Heliconius* is a diverse clade of brightly colored and chemically defended butterflies. This group is well-known for mimicry, in which different species evolve nearly identical wing patterns as a means of protection from predators [8]. Rapid evolution of wing pattern diversity in *Heliconius*, combined with convergence due to mimicry, has resulted in a group of closely related and hybridizing species, some of which look very different and others that look nearly identical. These two new papers show that alleles for wing patterning have moved across species boundaries multiple times, effectively transferring mimicry from one species to another.

This discovery of adaptive introgression in *Heliconius* builds upon five important prior advances. First, close to a decade ago, Larry Gilbert used results from a multitude of interspecific crosses to propose a model whereby *Heliconius* mimicry evolved by repeated interspecific transfer of color patterning alleles [9]. Second, surveys of wild-caught specimens have revealed many instances of natural hybridization in *Heliconius* [10], and molecular analyses based on neutral markers detected signatures of relatively widespread introgression among closely related species [11], [12]. Third, recent discoveries of cryptic species have provided multiple examples of sympatric, co-mimetic, and potentially hybridizing species [13]; species among which mimicry transfer might be particularly likely. Fourth, there has been a concerted effort by those studying *Heliconius* to map and characterize their mimicry loci [14]. This recently culminated in the identification of *optix* as the red patterning gene [15], with high-resolution SNP data revealing strong associations just upstream of the gene [16]. Finally, new population genetic data from *optix* itself revealed that, within polymorphic species like *H. erato* and *H. melpomene*, similar wing patterns in distinct subspecies share a common origin [17]. This result, while focused on within-species variation, showed that apparent convergence can result from shared ancestry of mimicry alleles.

The two new studies build on this foundation to explore adaptive introgression of mimicry using different but highly complementary approaches. Pardo-Diaz et al. [6] use amplicon sequencing from targeted portions around *optix*, combined with phylogenetic- and coalescent-based tests for gene flow. The Heliconius Genome Consortium [7] present a reference genome sequence for *H. melpomene*, and then use RAD markers, targeted resequencing, and an ABBA-BABA statistical approach to examine introgression genome-wide, with a special emphasis on the *optix* interval, as well as a second region that controls yellow color patterning. The results of the two studies are highly congruent—genetic variation from a narrow genomic interval just upstream of *optix* groups populations and species by red wing patterning rather than known phylogenetic relationships (Figure 1). Amazingly, this even applies to *H. elevatus*, a “rayed” pattern species from the silvaniform clade, a group that generally displays “tiger-stripe” patterns. Importantly, The Heliconius Genome Consortium [7] show that these signatures of introgression ultimately encompass hundreds of SNPs, ruling out the possibility that these groupings are the result of convergent molecular evolution.

**Figure 1:**
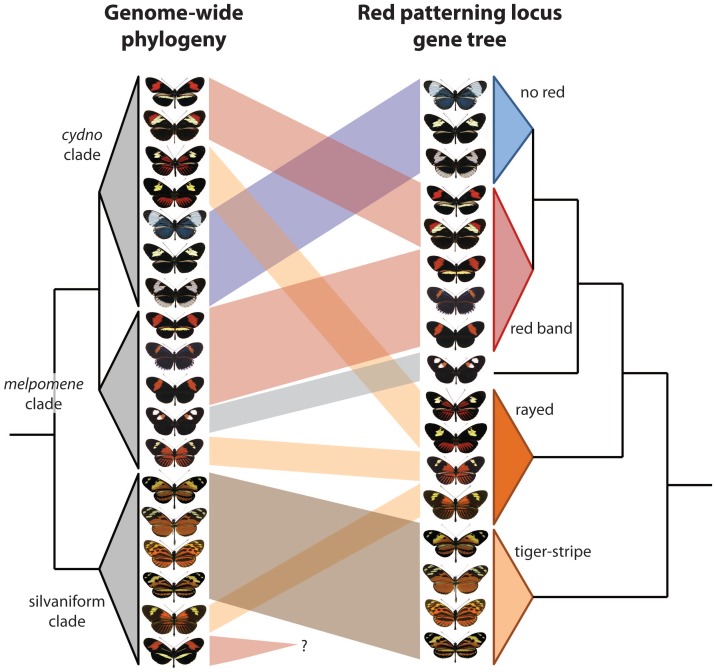
Two new papers show that wing patterning has been swapped among *Heliconius* butterfly species via introgressive hybridization. One clear signature of this history is the discordance between a phylogeny based on the entire genome and one based on a single portion of the genome that controls wing patterning, in this case an intergenic region near *optix* that is strongly associated with red patterning [15], [16]. The genome-based tree (left) reflects the known organismal phylogeny, while the *optix*-based tree groups individuals by phenotype. This figure, which consolidates the findings of Pardo-Diaz et al. [6] and The Heliconius Genome Consortium [7], depicts only a subset of the species and wing pattern phenotypes contained in the *melpomene*/*cydno*/silvaniform clade of *Heliconius*. Furthermore, this clade is but one of four major clades within the genus.

This evidence for adaptive introgression is striking, yet there is much that remains unknown. For instance, *H. besckei* is the only silvaniform species with a “red band” phenotype, but it remains unknown if it swapped mimicry genes with other red banded species (Figure 1). Furthermore, there are potential instances of more subtle introgression that have yet to be explored; cases in which a single pattern element, as opposed to an entire phenotype, appears to be shared between species [9]. Other important questions include: In which species did mimicry alleles originally arise and when did they spread? How frequently does mimicry introgression precipitate speciation? How do these closely related, sympatric, co-mimetic taxa remain distinct given color pattern's important role in generating reproductive isolation [18]? What about adaptive introgression of other traits and genes not related to mimicry? Has introgression contributed to diversity and mimicry in other *Heliconius* clades?

Beyond *Heliconius*, it is important that we better understand the frequency and taxonomic distribution of adaptive introgression, as well as its potential link to adaptive radiation. These new results from *Heliconius* reveal that introgression has played an essential role in driving adaptive evolution across an entire clade. Whether we find a more general role for introgression in facilitating adaptive radiation remains to be seen. However, the fact that the first comprehensive examination revealed widespread adaptive introgression certainly makes this a very real possibility.

## References

[1] Arnold ML, Martin NH (2009). Adaptation by introgression.. J Biol.

[2] Kunte K, Shea C, Aardema ML, Scriber JM, Juenger TE (2011). Sex chromosome mosaicism and hybrid speciation among tiger swallowtail butterflies.. PLoS Genet.

[3] Burke JM, Arnold ML (2001). Genetics and the fitness of hybrids.. Annu Rev Genet.

[4] Song Y, Endepols S, Klemann N, Richter D, Matuschka FR (2011). Adaptive introgression of anticoagulant rodent poison resistance by hybridization between old world mice.. Curr Biol.

[5] Seehausen O (2004). Hybridization and adaptive radiation.. Trends Ecol Evol.

[6] Pardo-Diaz C, Salazar C, Baxter SW, Merot C, Figueiredo-Ready W (2012). Adaptive introgression across species boundaries in *Heliconius* butterflies.. PLoS Genet.

[7] The Heliconius Genome Consortium (2012). Butterfly genome reveals promiscuous exchange of mimicry adaptations among species.. Nature.

[8] Müller F (1879). *Ituna* and *Thyridia*; a remarkable case of mimicry in butterflies.. Trans Entomol Soc Lond.

[9] Gilbert LE, Boggs CL, Watt WB, Ehrlich PR (2003). Adaptive novelty through introgression in *Heliconius* wing patterns: evidence for shared genetic “tool box” from synthetic hybrid zones and a theory of diversification.. Ecology and evolution taking flight: butterflies as model systems.

[10] Mallet J, Beltran M, Neukirchen W, Linares M (2007). Natural hybridization in heliconiine butterflies: the species boundary as a continuum.. BMC Evol Biol.

[11] Bull V, Beltran M, Jiggins CD, McMillan WO, Bermingham E (2006). Polyphyly and gene flow between non-sibling *Heliconius* species.. BMC Biol.

[12] Kronforst MR, Young LG, Blume LM, Gilbert LE (2006). Multilocus analyses of admixture and introgression among hybridizing *Heliconius* butterflies.. Evolution.

[13] Giraldo N, Salazar C, Jiggins CD, Bermingham E, Linares M (2008). Two sisters in the same dress: *Heliconius* cryptic species.. BMC Evol Biol.

[14] Papa R, Martin A, Reed RD (2008). Genomic hotspots of adaptation in butterfly wing pattern evolution.. Current Opin Gen Dev.

[15] Reed RD, Papa R, Martin A, Hines HM, Counterman BA (2011). *optix* drives the repeated convergent evolution of butterfly wing pattern mimicry.. Science.

[16] Nadeau NJ, Whibley A, Jones RT, Davey JW, Dasmahapatra KK (2012). Genomic islands of divergence in hybridizing *Heliconius* butterflies identified by large-scale targeted sequencing.. Phil Trans R Soc B.

[17] Hines HM, Counterman BA, Papa R, Albuquerque de Moura P, Cardoso MZ (2011). Wing patterning gene redefines the mimetic history of *Heliconius* butterflies.. Proc Natl Acad Sci U S A.

[18] Jiggins CD, Naisbit RE, Coe RL, Mallet J (2001). Reproductive isolation caused by colour pattern mimicry.. Nature.

